# Comparative effectiveness studies to improve clinical outcomes in end stage renal disease: the DEcIDE patient outcomes in end stage renal disease study

**DOI:** 10.1186/1471-2369-13-167

**Published:** 2012-12-06

**Authors:** Ebony L Boulware, Navdeep Tangri, Patti L Ephraim, Julia J Scialla, Stephen M Sozio, Deidra C Crews, Tariq Shafi, Dana C Miskulin, Jiannong Liu, Wendy St Peter, Bernard G Jaar, Albert W Wu, Neil R Powe, Sankar D Navaneethan, Karen Bandeen-Roche

**Affiliations:** 1 Welch Center for Prevention, Epidemiology and Clinical Research, Johns Hopkins Medical Institutions, 2024 E. Monument Street, Suite 2-600, Baltimore, MD, 21205, USA; 2 Division of General Internal Medicine, Johns Hopkins School of Medicine, Baltimore, MD, USA; 3 Division of Nephrology, University of Manitoba, Manitoba, Canada; 4 Department of Epidemiology, Johns Hopkins Bloomberg School of Public Health, Baltimore, MD, USA; 5 Division of Nephrology, University of Miami Miller School of Medicine, Miami, FL, USA; 6 Division of Nephrology, Johns Hopkins University School of Medicine, Baltimore, MD, USA; 7 Division of Nephrology, Tufts University School of Medicine, 136 Harrison Avenue, Boston, MA, 02111, USA; 8 Chronic Disease Research Group, 914 South 8th Street, Minneapolis, MN, USA; 9 University of Minnesota, College of Pharmacy, 308 Harvard St. SE, Minneapolis, MN, 55455, USA; 10 Nephrology Center of Maryland, 5601 Loch Raven Boulevard, Baltimore, MD, 21239, USA; 11 Department of Health Policy and Management, Johns Hopkins Bloomberg School of Public Health, Baltimore, MD, USA; 12 San Francisco General Hospital and University of California, San Francisco, 1001 Potrero Avenue, San Francisco, CA, 94110, USA; 13 Department of Nephrology and Hypertension, Glickman Urological and Kidney Institute, Cleveland Clinic, 9500 Euclid Avenue, Cleveland, OH, 44195, USA; 14 Department of Biostatistics, Johns Hopkins Bloomberg School of Public Health, Baltimore, MD, USA

## Abstract

**Background:**

Evidence is lacking to inform providers’ and patients’ decisions about many common treatment strategies for patients with end stage renal disease (ESRD).

**Methods/design:**

The DEcIDE Patient Outcomes in ESRD Study is funded by the United States (US) Agency for Health Care Research and Quality to study the comparative effectiveness of: 1) antihypertensive therapies, 2) early versus later initiation of dialysis, and 3) intravenous iron therapies on clinical outcomes in patients with ESRD. Ongoing studies utilize four existing, nationally representative cohorts of patients with ESRD, including (1) the Choices for Healthy Outcomes in Caring for ESRD study (1041 incident dialysis patients recruited from October 1995 to June 1999 with complete outcome ascertainment through 2009), (2) the Dialysis Clinic Inc (45,124 incident dialysis patients initiating and receiving their care from 2003–2010 with complete outcome ascertainment through 2010), (3) the United States Renal Data System (333,308 incident dialysis patients from 2006–2009 with complete outcome ascertainment through 2010), and (4) the Cleveland Clinic Foundation Chronic Kidney Disease Registry (53,399 patients with chronic kidney disease with outcome ascertainment from 2005 through 2009). We ascertain patient reported outcomes (i.e., health-related quality of life), morbidity, and mortality using clinical and administrative data, and data obtained from national death indices. We use advanced statistical methods (e.g., propensity scoring and marginal structural modeling) to account for potential biases of our study designs. All data are de-identified for analyses. The conduct of studies and dissemination of findings are guided by input from Stakeholders in the ESRD community.

**Discussion:**

The DEcIDE Patient Outcomes in ESRD Study will provide needed evidence regarding the effectiveness of common treatments employed for dialysis patients. Carefully planned dissemination strategies to the ESRD community will enhance studies’ impact on clinical care and patients’ outcomes.

## Background

End stage renal disease (ESRD) is a significant and growing public health problem with increasing incidence and prevalence in the United States and worldwide [1]. Patients with ESRD have rates of cardiovascular, cerebrovascular, and infectious morbidity that are up to several times greater than people in the general population, as well as worse quality of life [2-10]. As a result, the cost of caring for patients with ESRD is exceedingly high, amounting for over $21 billion total in 2007 and approximately 6% of total Medicare expenditures [1].

Despite the intensity and cost of care for patients with ESRD, little is known about the impact of many common clinical management strategies that are intended to improve health outcomes in ESRD [11]. In the absence of rigorous evidence to guide clinical practice, many strategies employed to treat common illnesses in the general public have been adapted to treat the ESRD population, with unclear effects on patients’ clinical outcomes [11-18]. As a result, clinicians and patients are unsure about which of these strategies are best. Rigorous research on the effectiveness of common treatment strategies for patients with ESRD could help improve decision-making, the quality of ESRD care and patients’ health.

As part of its Effective Health Care Program, the Agency for Healthcare Research and Quality (AHRQ) supports the Developing Evidence to Inform Decisions about Effectiveness (“DEcIDE”) Network to conduct “studies on the outcomes, effectiveness, safety and usefulness of medical treatments and services” [19]. In 2009, the Effective Health Care Program received funding through the American Recovery and Reinvestment Act (ARRA) to enhance its’ efforts to perform high-quality comparative effectiveness studies in priority areas. [20] The DEcIDE Patient Outcomes in End Stage Renal Disease Study was commissioned with these funds to better understand the comparative effectiveness of different, commonly employed treatment strategies on improving cardiovascular disease (CVD) and other important health outcomes among patients with ESRD, including: (1) antihypertensive medication regimens, (2) initial timing of dialysis initiation, and (3) iron management strategies. In this paper, we describe the general approach to comparative effectiveness studies employed in the DEcIDE Patient Outcomes in End Stage Renal Disease Study.

## Methods/design

The study is being conducted through collaboration of researchers at five centers (Johns Hopkins University (coordinating center), Tufts University Medical Center, Minneapolis Medical Research Foundation, the University of California, San Francisco, and the Cleveland Clinic Foundation), which are conducting several observational “sub-studies” addressing the three comparative effectiveness questions identified by AHRQ for investigation (aims and hypotheses in Table 1). Sub-studies draw data from up to four nationally representative linked data sources capturing the clinical care and outcomes of patients with chronic kidney disease (CKD) or ESRD and utilize rigorous methodology to compare the effectiveness of kidney disease treatment strategies. Data sources provide primary data (e.g., from chart reviews and from patient report), treatment-level clinical data (e.g., extracted from electronic health records), and insurance claims (e.g., hospitalization dates) collected in the course of CKD and dialysis care.

**Table 1:** Specific aims of sub-studies in the DEcIDE Patient Outcomes in End Stage Renal Disease Study

**Sub-Study**	**Specific Aim**
1. Antihypertensive management	a. To assess the comparative effectiveness of ACE inhibitors/ARBs , Beta Blockers, and Calcium Channel Blockers in preventing all cause and cardiovascular disease (CVD) mortality among dialysis patients
	b. To assess the comparative effectiveness of ACE inhibitors/ARBs, Beta Blockers and Calcium Channel Blockers in preventing morbidity (CVD events, all cause hospitalizations, quality of life, worsening disability) among patients on dialysis
2. Timing of dialysis initiation	a. To assess the comparative effectiveness of early versus conventional dialysis initiation on risk of all cause and cardiovascular mortality
	b. To assess the comparative effectiveness of early versus conventional dialysis initiation on morbidity (cardiovascular events, all cause and infectious hospitalizations, health-related quality of life, comorbid disease control, and nutritional status)
3. Intravenous iron for anemia management	a. To compare the effectiveness of iron dosing strategies (regular maintenance therapy versus intermittent ‘as needed’ therapy) on anemia management
	b. To assess the comparative effectiveness of lower versus higher cumulative IV iron doses on CVD-related hospitalization and mortality, infection-related hospitalization and mortality, all cause mortality, and quality of life

Consistent with the goals of comparative effectiveness research to perform studies that are relevant to decision-makers in ESRD care, we have engaged an Expert Stakeholder Group representing key decision-makers (including patients, health care providers, policy makers, and regulators) within the ESRD community in an ongoing fashion.

### Common data sources

Four main data sources from which sub-studies derive their data comprise representative US patient populations with kidney disease that are followed through active and passive means until death. Cohorts are also linked to (or are composed of) registry data and Centers for Medicare and Medicaid Services (CMS) claims generated during the course of ESRD care. Data are linked to national death data sources to ascertain patients’ vital status and underlying cause of death. (Tables 2 and 3).

**Table 2:** Characteristics of data sources employed in the DEcIDE Patient Outcomes in End Stage Renal Disease Study

**Data Source**	**Participants**	**Years**	**Setting**	**Data Collection**	**Linkages**
National registry (USRDS*)	428,411 incident dialysis patients	2006 -2009	Retrospectively identified cohort	Medicare Claims	National Death Files; Medicare Part D prescription drug data
Dialysis Clinic, Inc. (DCI)	45,124 incident dialysis patients	2003 -2010	Retrospectively identified cohort	Clinical Data	National Registry (USRDS); National Death Files; Medicare Part D prescription drug data
Choices for Healthy Outcomes in Caring for Patients with ESRD (CHOICE)	1,041 incident dialysis patients	1995 -2009	Prospective Cohort Study	Primary Collection of Clinical Data, Questionnaires	National Registry (USRDS); National Death Files
Cleveland Clinic Foundation Chronic Kidney Disease Registry	53,399 patients with chronic kidney disease	2005-2009	Retrospectively identified cohort	Clinical Data	National Registry (USRDS); National Death Files

*USRDS=United States Renal Data System.

**Table 3:** Sample data elements of data sources

**Data Element Type**	**Data Source**			
	**CHOICE**	**DCI**	**USRDS Registry**	**Cleveland Clinic CKD Registry**
**Demographics**	Present	Present	Present	Present
**Outpatient Medications**	Medical records	Medical records	Medicare Part D prescription drug data Claims 2006-2009	Medical records
**Facility/Intravenous Medications**	Medical records and Claims	Medical records and Claims	Claims	Medical records and Claims
**Laboratory Data**	Clinical Data	Clinical Data	Not Available	Clinical Data
**Quality of Life**	Detailed Disease Specific and Global	Global	Not Available	Not Available
**Co-morbidity**	Disease Specific Scale	Medical Records	Claims	Medical Records
**Hospitalizations**	Medical Records	Medical Records	Claims	Medical Records
**CVD Events**	Adjudicated from medical records, claims	Claims	Claims	Not Available
**Deaths**	Medical records, Medicare enrollment, National Death Index	Medicare enrollment, National Death Index	Medicare enrollment, National Death Index	Social Security Death Index

#### Choices for healthy outcomes in caring for End-stage renal disease (CHOICE) study

CHOICE is a longitudinal observational cohort study of 1,041 incident dialysis patients originally funded by AHRQ to measure several aspects of patients’ experiences and outcomes related to their choice of renal replacement therapy [21]. CHOICE includes a highly demographically diverse sample of patients cared for around the US in urban and rural settings, and receiving peritoneal dialysis or in-center hemodialysis. The CHOICE population (initial enrollment in 1995, with ongoing passive follow-up) has more detailed information about comorbidity, laboratory data, process of care, and clinical outcomes than registries and other retrospective data sources, enhancing its benefits for use in effectiveness studies [22]. CHOICE data are linked to the USRDS registry to obtain information on patients’ health conditions and health care utilization. CHOICE data are also linked to the National Death Index to confirm patients’ vital status and cause-specific mortality, which were confirmed with comprehensive chart reviews.

#### Dialysis Clinic, Inc (DCI)

DCI is the largest non-profit provider of dialysis care and third largest dialysis provider in the US, serving approximately 13,000 patients at 209 dialysis facilities in 27 states. The population of patients within DCI is diverse and is demographically representative of the US dialysis population, with an overrepresentation of African Americans [23]. Information about comorbid conditions, hospitalization dates, oral and intravenous medications, quality of life, the delivered dialysis prescription, as well as physician, nurse, dietician and social worker progress notes are entered into an electronic medical record. Researchers have used data from DCI to perform a variety of studies assessing quality of care and clinical outcomes in patients with ESRD, including studies of anemia management, adherence to clinical practice guidelines, and factors affecting important clinical outcomes [24,25]. We have obtained data on all incident patients within DCI from 2003–2010 and we have linked data to the USRDS registry to obtain information on patients’ health conditions and health care utilization.

#### United States Renal Data System (USRDS) and Centers for Medicare and Medicaid Services (CMS)

The United States Renal Data System (USRDS) is a national data system (funded by the National Institute of Diabetes and Digestive and Kidney Diseases) that collects, analyzes, and distributes information about ESRD in the United States. USRDS collaborates with the CMS, the United Network for Organ Sharing (UNOS), and the ESRD networks to share datasets. These data comprise several sub-files which, when linked, provide patient-level data on a) demographics, primary diagnoses, limited comorbidities and biochemical tests at dialysis initiation; b) health care services utilization and intravenous medications delivered in dialysis facilities (Form 2728); c) deaths, including cause of death (Form 2846) and d) outpatient medication use in patients receiving benefits through Medicare Part D prescription drug program [26]. These data also include facility-level data regarding facility characteristics and CMS performance metrics for all patients receiving ESRD care under Medicare [1]. We have constructed a cohort of all US patients initiating dialysis from 2006–2009. For members of the cohort who initiated dialysis after age 65 and had enrolled in Medicare prior to initiating dialysis, we have also obtained up to two years of pre-dialysis CMS claims which provides additional ascertainment of patient comorbidity and health care services utilization before the initiation of dialysis. We have linked this cohort to the National Death Index to confirm patients’ vital status and cause-specific mortality.

#### Cleveland Clinic Foundation Chronic Kidney Disease Registry

The Cleveland Clinic Foundation Registry contains clinical data on over 53,000 patients with CKD based on estimated glomerular filtration rate (eGFR) or administrative billing codes obtained through clinical care in the Cleveland Clinic health care system. Data extracted from patients’ electronic health records include information on patients’ comorbid conditions, laboratory values, and health care services utilization. These data have been linked to the National Social Security Death Index as well as the USRDS data, providing additional information on patients’ vital status as well as their ESRD incidence, and ESRD-related health and health care utilization [27].

### Commonly defined variables

When possible, sub-studies utilize commonly defined variables to assess patient demographic variables and comorbid conditions ascertained at studies’ baseline. Studies also attempt to incorporate similarly defined morbidity and mortality outcomes and censoring rules in main analyses.

#### Patient demographics

We ascertain patient demographics (age, gender, race, education) through both administrative (from USRDS CMS-2728 form) and clinical data, where available. In CHOICE, some demographic data collected directly from the participants are available [22,28].

#### Comorbid conditions

We utilize validated Medicare claims-based measures to assess the presence and severity of patients’ comorbid illnesses at the time of dialysis initiation and throughout the course of treatment [29].

#### Mortality

We ascertain all-cause mortality and cause-specific mortality using cause of death information classified according to the 10^th^ revision of the International Classification of Disease (ICD-10) obtained from the National Death Index, which is commonly used to ascertain date and cause of death in population-based studies [30]. We compare information on cause of death in the USRDS data to information on cause of death collected from dialysis providers as part of the USRDS registry (i.e., ESRD Death Notification, Form CMS-2746) [31]. (Additional file 1)

#### Morbidity

We ascertain infectious and CVD events (diagnoses and hospitalizations) and other morbidity (including all cause hospitalizations and health-related quality of life) using Medicare and clinical data, where available. We will obtain data on all hospitalizations from Medicare claims for all three data sources. We will employ validated definitions using ICD9-CM codes to determine infectious and cardiovascular hospitalizations. Subcategories of cardiovascular events (ischemic, heart failure and arrhythmia related) will be explored. Health-related quality of life data are also available in CHOICE and DCI [32]. **(**Additional file 1**)**

### Common approaches to statistical analyses

A primary challenge of observational comparative effectiveness research is to produce inferences that have plausibly causal interpretations in order to simulate randomized effectiveness trials. The state of the art for addressing this challenge is considered by many to be methods employing inverse probability weighting to develop analytic cohorts whose characteristics and prior states are balanced across treatment groups, such as inversely-weighted propensity scoring [33,34] and marginal structural modeling, which we will employ in primary analyses [35]. However, because such methods can exhibit sensitivity to extremes among the weights, we will also perform sensitivity analyses within and across data sources to validate main findings and contrast inversely probability weighted analyses with generalized estimating equations [36] based analyses. The latter will incorporate covariate adjustment to predict treatment selection with independence working correlation structure and robust variance correction so as to avoid propagation of endogeneity [37]. Primary outcomes are times-to-events; to make feasible the analytic approaches just described, analyses will be implemented in fine-grain discrete time (i.e., at intervals of 1, 3, or 6 months) [38].

Analyses will address additional challenges, including clustering of data within providers, competing and semi-competing risks (e.g. transplantation, death) [38], effective summarization of treatment history (e.g., iron dosing over time), and informative missing data. Analyses for relative effectiveness of early and later dialysis initiation pose challenges of immortal time bias [39] and indeterminate “origin” for time-to-event analyses (i.e. age at which a given disease severity is reached that makes a person a reasonable candidate for dialysis). We are addressing these challenges through the application of inverse probability weighted analyses [40].

### Stakeholder and external technical expert engagement panels

We have engaged stakeholders from the ESRD community representing patients, providers, payers, and regulators to ensure the relevance of our sub-study questions and approach, and to help guide dissemination of our findings. (Table 4) Stakeholders include patients with kidney disease, a representative from the major dialysis providers, health care insurers, and representatives from federal agencies involved in monitoring ESRD treatment. In addition, we have engaged federal agencies involved in funding studies in ESRD. We obtained stakeholders’ input to modify our preliminary analysis, for example, expanding our analyses to explore the long-term end organ toxicity of iron and the effect of patients’ cardiovascular volume status on effectiveness of antihypertensive agents. As of date, we have convened 3 face-to-face stakeholder meetings and will engage stakeholders continuously throughout the project.

**Table 4:** Expert Stakeholder Advisory Group

**Stakeholder**	**Organization Represented**
Healthcare Providers	American Society of Nephrology, US Department of Veterans Affairs
National Consumer Organizations	National Kidney Foundation, American Kidney Fund
Federal Government	National Institute of Diabetes and Digestive and Kidney Diseases, National Heart, Lung and Blood Institute, Food and Drug Administration
Renal Replacement Therapy Patients	Transplant Recipient, Patient receiving Home Hemodialysis
Dialysis Provider	Dialysis Clinic Inc.
Payers/Regulators	Centers for Medicare and Medicaid Services, Mid-Atlantic Renal Coalition, United Health Care, Johns Hopkins Health Care, CIGNA

## Approach to Sub-studies

### Sub-studies comparing effectiveness of antihypertensive therapies

We are studying the effectiveness of three major antihypertensive medication classes on patients’ morbidity, mortality and quality of life. Our analyses will account for several relevant potential confounders and mediators of the association of antihypertensive medication therapy choice with patient outcomes, including blood pressure control, underlying cardiovascular disease and other comorbidities, and patients’ cardiovascular volume status.

#### Antihypertensive therapy

We are assessing whether patients are receiving (either alone or in combination) one of three major classes of antihypertensive medications: (a) angiotensin converting enzyme (ACE) inhibitors or angiotensin II receptor blocking agents (ARBs); (b) beta receptor blocking agents; or (c) calcium channel blocking agents. We are utilizing data from both clinical sources (i.e., medications abstracted from chart review and dialysis facility medication lists) and administrative claims (i.e., Medicare Part D data) to ascertain doses of these medications prescribed to patients over time, as well as medication adherence [26]. We are assessing changes in medications on a monthly basis, and we are also assessing the presence of non-standard treatment regimens, including regimens incorporating medication administration on alternate days and “PRN” or as-needed orders for antihypertensive therapies.

#### Other Key exposures and correlates

We are measuring other variables which may influence antihypertensive treatment patterns and effectiveness of antihypertensive therapy, including: (1) blood pressure and blood pressure variability, (2) patient volume status, (3) dialysis dose, (4) dialysis adherence, and (4) the presence of influential comorbid conditions such as cardiovascular disease and congestive heart failure.

#### Study cohorts

Study cohorts allow for descriptions of individual patient changes in anti-hypertension therapy regimens over time as well as to assess the influence of antihypertensive regimens and changes in antihypertensive regimens on proximal and distal outcomes. We have created analytic datasets assessing patient time in discrete time intervals, including 30, 60, and 90 day windows. Key exposure variables and confounders are assessed within discrete time intervals, allowing for time their varying assessment.

#### Analytic strategies

Our main analyses attempt to identify the comparative effectiveness of distinct antihypertensive regimens (e.g. beta-blocker versus ACE or ARB containing regimens) on outcomes in longitudinal models. We are utilizing discrete time proportional hazards models to study the association of time-varying exposures and correlates with outcomes as well as marginal structural models, which provide an opportunity to account for time dependent influences of confounders on anti-hypertension treatment choice through inverse probability weighting techniques. Our analyses also attempt to capture differences in the effectiveness of antihypertensive therapies on potential acute (e.g., prevention of arrhythmias or sudden cardiac death) versus more chronic (development of cardiovascular disease) outcomes by incorporating varying lag times (ranging from no window a 90 day window) between assessments of exposures and outcomes.

#### Examples of challenges faced

Defining patients’ blood pressure regimens represents a key challenge. In exploratory analyses, we identified over 100 distinct combinations of blood pressure medications used by patients receiving care in DCI. Many patients received more than one of the key antihypertensive therapies of interest, while others received numerous blood pressure combination regimens that changed over time. In order to provide decision makers with evidence they will deem usable, we will simplify our definitions of regimens to ensure they directly address key decisions clinicians would confront commonly in clinical practice (e.g., a decision to implement a regimen that contains a beta blocker versus a regimen that contains an ACE inhibitor or a regimen that contains both agents).

### Sub-studies comparing effectiveness of early versus later dialysis initiation

We are studying the effects of starting dialysis early versus later on patients’ of morbidity, mortality, and quality of life outcomes. Using data collected prior to patients’ dialysis initiation when possible, we will account for patients’ clinical conditions that could lead to earlier dialysis initiation (e.g., fluid overload from congestive heart failure). We also seek to account for the contribution of local or regional practice variations in dialysis care [23,41].

#### Early versus later timing of dialysis initiation

We define early versus later dialysis initiation based on pre-defined thresholds of estimated glomerular filtration rate (eGFR) (e.g., eGFR greater than 10ml/min/1.73m^2^ versus less than 10ml/min/1.73m^2^). We obtain (when available) information on GFR at dialysis initiation from provider questionnaires (i.e., obtained from USRDS CMS form 2728). For analyses among patients with pre-dialysis CKD (i.e., stage 4 CKD, in the Cleveland Clinic registry), we obtain information on eGFR from laboratory records extracted from patients’ electronic health records.

#### Other Key exposures and correlates

We are also measuring other variables that may influence patients’ need for early dialysis, including the burden and severity of comorbid illnesses (e.g., congestive heart failure) and the occurrence of medical events (e.g., hospitalizations) in the pre-dialysis period. When data are available, we also assess the frequency of patients’ utilization of pre-dialysis nephrology care and their preparation for renal replacement therapy (e.g., fistula placement) prior to dialysis initiation.

#### Construction of study cohorts

We are constructing cohorts to allow for descriptions of pre-dialysis factors influencing the initiation of early dialysis when possible. This includes a cohort of patients initiating dialysis who have pre-dialysis CMS claims linked to their CMS dialysis health claims (in USRDS) as well as a cohort of patients seen in nephrology clinics who have dialysis CMS claims linked to their pre-dialysis health records (in Cleveland Clinic). In cases where pre-dialysis claims are not available, we are using data collected from physicians at the time of patients’ dialysis initiation (i.e., from CMS-2728 form).

#### Analytic strategies

Our analyses address limitations in previous observational studies of the comparative effectiveness of early versus later dialysis initiation [42-46]. One key limitation of prior studies is their inability to account for differences in patients’ pre-dialysis health and health care utilization which may have influenced their initiation of dialysis. We strive to construct cohorts with information on patients’ pre-dialysis health and health care utilization whenever possible. We are employing propensity score methods and instrumental variables to account for observed and unobserved cofounders previously observed to predict between early dialysis initiation and poor outcomes. We are also using newly developed modeling techniques [47] to address biases (e.g., immortal person time) associated with observing patients only from the time of their earlier versus later dialysis initiation to outcomes.

#### Example of challenges faced

A key challenge we face in constructing cohorts is the problem of adequately capturing patients’ pre-dialysis health and trajectory of CKD progression prior to dialysis initiation. For instance, patients may suffer from symptoms (e.g., extreme fatigue or weight loss due to poor appetite) that could heavily influence patients’ and providers’ preferences for initiating dialysis. To address this challenge, we are attempting to capture pre-dialysis comorbidity by utilizing datasets that collect information on pre-dialysis health status as assessed in billing data and medical records. We are adapting previously validated comorbidity instruments, and trying to capture measures of the frequency or intensity of health care utilization (e.g., number pre-dialysis hospitalizations), which we hypothesize could reflect patients’ health declines and perceived need for earlier dialysis.

### Sub-studies comparing effectiveness of anemia management strategies

We are studying the safety and effectiveness of various iron dosing strategies in the care of dialysis patients. We will consider both the risk of infectious morbidity and mortality related to iron administration as well as the risk of end-organ damage (i.e., from iron deposition). We will assess effectiveness of various dosing strategies in terms of the achievement of hemoglobin treatment goals [48], erythropoietin stimulating agent use and the frequency of blood transfusions.

#### Iron administration

We are assessing cumulative iron utilized over short and longer time periods as well as the pattern of iron administration (e.g., intermittent bolus administration versus maintenance dosing). We will ascertain iron doses from both clinical data (i.e., medications abstracted from electronic chart review and dialysis facility medication lists) and administrative billing data (i.e., dialysis intravenous medication claims from CMS).

#### Other Key exposures and correlates

We are measuring other variables that may influence iron dosing patterns and effectiveness of iron therapy, including (1) the presence of inflammation or infection, (2) patient comorbidity, (3) use of other medications to treat anemia (i.e., erythropoiesis stimulating agents), and (4) patients’ response to anemia therapy.

#### Construction of study cohorts

We are constructing study cohorts to allow for descriptions of individual patient changes in iron therapy regimens over time as well as to assess the influence of iron dose and changes in dosing regimens on proximal and distal outcomes. We have created analytic datasets assessing patient time in discrete time intervals, including 30, 60, 90, and 180 day windows. Key exposure variables and confounders are assessed within discrete time intervals, allowing for their time varying assessment.

#### Analytic strategies

Our main analyses will compare the effectiveness of varying levels of iron dose on outcomes in longitudinal models. We are utilizing discrete time proportional hazards models to study the association of time-varying exposures and correlates with outcomes as well as marginal structural models, which provide an opportunity to account for time dependent influences of confounders on iron therapy. Our analyses will assess differences in the effectiveness of iron therapies on acute (e.g., infectious outcomes) and more chronic (e.g., cardiovascular disease) outcomes by incorporating varying lag times (ranging from no window a 90 day window) between assessments of exposures and outcomes.

#### Example challenges

Evaluating the short versus longer-term toxicity of iron presents a challenge. While cardiovascular events and iron deposition disorders may represent intermediate and long term outcomes, acute infectious hospitalizations may be proximally related to iron administration. Our analyses will calculate total iron exposure over varying discrete time periods (i.e., 30, 60, 90, or 180 days) and incorporate a time lag of up to 30 days to help distinguish short versus long-term effect of iron on outcomes. Another challenge is the incorporation of laboratory values (e.g., transferrin saturation or ferritin), which are measured at infrequent intervals but which influence physicians’ administration of iron. We have created longer rolling ‘windows’ over which we observe whether these laboratory values have been obtained, identifying both average values as well as most recently obtained values as they may be most influential on physicians’ iron prescribing patterns.

### Ethics and dissemination of study findings

All data sources are de-identified prior to analyses. We are performing all analyses of USRDS, CHOICE, and DCI data with the approval of the Johns Hopkins Institutional Review Board. Analyses of Cleveland Clinic data are performed at the Cleveland Clinic with approval of the Cleveland Clinic Institutional Review Board. We will work with our Stakeholder Panel to provide insight into optimal avenues and formats (e.g., trade publications, social media) for dissemination of our findings. We will also work with the Eisenberg Center within AHRQ to disseminate our study findings widely among stakeholders in the ESRD community.

## Discussion

There is a great need for studies comparing the effectiveness of common treatment strategies employed in the care of patients with ESRD. The DEcIDE Patient Outcomes in End Stage Renal Disease Study addresses questions commonly confronted by decision makers (including patients, health care providers, payers and policy-makers) involved in ESRD care.

Several aspects of the DEcIDE Patient Outcomes in End Stage Renal Disease Study distinguish it from other ongoing observational studies. First, our use of clinical and administrative data will facilitate our study of actual clinical practice patterns, enhancing the relevance of our findings to decision makers. Second, our data incorporate both clinical and administrative information, enhancing the specificity with which we can classify key exposures, confounders, and outcomes. Examination of four complementary cohorts will enable triangulation of results. If consistent findings are obtained, this will enhance decision makers’ confidence to act on evidence emerging from our studies. Third, our continuous engagement of stakeholders throughout the conduct of the study is novel among ESRD studies, and will enhance how the ESRD community values and integrates our findings. Fourth, our nationally representative cohorts feature some data linkages that have not previously been performed in comparative effectiveness studies among ESRD patients. Studies previously assessing cause specific mortality in ESRD have relied on dialysis physician reports in CMS reports (Form 2746). Our data linkages with the National Death Index for determination of cause specific mortality will help us validate these reports and may enhance the comparability of our findings to epidemiological studies in other areas. Finally, our use of novel strategies to overcome limitations (e.g., immortal person time in assessments of early versus later dialysis initiation) of prior studies may help to better inform previously unanswered questions in ESRD care.

In conclusion, the DEcIDE Patient Outcomes in End Stage Renal Disease Study is funded to address gaps in evidence regarding the effectiveness of common treatment strategies employed in ESRD care. Study of these strategies in four representative US cohorts of patients with kidney disease using rigorous statistical methodology and ongoing engagement of stakeholders in the ESRD community enhances the likelihood these studies will yield findings deemed relevant and useful to decision makers in ESRD care.

## Abbreviations

AHRQ: Agency for Healthcare Research and Quality; DEcIDE: the Developing Evidence to Inform Decisions about Effectiveness; ESRD: End stage renal disease; ARRA: American Recovery and Reinvestment Act; CVD: Cardiovascular disease; CKD: Chronic kidney disease; CHOICE: Choices for Healthy Outcomes in Caring for End-Stage Renal Disease Study; DCI: Dialysis Clinic, Inc; USRDS: United States Renal Data System; CMS: Centers for Medicare and Medicaid Services; UNOS: United Network for Organ Sharing; eGFR: Estimated glomerular filtration rate; ACE: Angiotensin converting enzyme; ARB: Angiotensin II receptor blocking agent.

## Competing interests

This project is supported by the DEcIDE Network AHRQ contract number HHSA290200500341I, Task Order #6. Dr. Shafi was supported by K23DK083514 from the National Institute of Diabetes and Digestive and Kidney Diseases.

AHRQ Disclosure: Identifiable information, on which this report, presentation, or other form of disclosure is based, is confidential and protected by federal law, Section 903(c) of the Public Health Service Act, 42 USC 299a-1(c). Any identifiable information that is knowingly disclosed is disclosed solely for the purpose for which it has been supplied. No identifiable information about any individual supplying the information or described in it will be knowingly disclosed except with the prior consent of that individual. The authors declare that they have no competing interests.

## Authors’ contributions

**LEB** contributed to the conception and design of the studies, analyses and data interpretation, drafting the manuscript, and has approved the final version for publication. **NT** contributed to the conception and design of the studies, analyses and data interpretation, drafting the manuscript, and has approved the final version for publication. **PLE** contributed to the conception and design of the studies, led the data acquisition, contributed to analyses and data interpretation, drafting the manuscript, and has approved the final version for publication. **JS** contributed to the conception and design of the studies, analyses and data interpretation, drafting the manuscript, and has approved the final version for publication. **SS** contributed to the conception and design of the studies, analyses and data interpretation, drafting the manuscript, and has approved the final version for publication. **DCC** contributed to the conception and design of the studies, analyses and data interpretation, drafting the manuscript, and has approved the final version for publication. **TS** contributed to the conception and design of the studies, analyses and data interpretation, drafting the manuscript, and has approved the final version for publication. **DM** contributed to the conception and design of the studies, analyses and data interpretation, drafting the manuscript, and has approved the final version for publication. **JL** contributed to the conception and design of the studies, analyses and data interpretation, drafting the manuscript, and has approved the final version for publication. **WLSP** contributed to the conception and design of the studies, analyses and data interpretation, drafting the manuscript, and has approved the final version for publication. **BGJ** contributed to the conception and design of the studies, analyses and data interpretation, drafting the manuscript, and has approved the final version for publication. **AW** contributed to the conception and design of the studies, analyses and data interpretation, drafting the manuscript, and has approved the final version for publication. **NRP** contributed to the conception and design of the studies, analyses and data interpretation, drafting the manuscript, and has approved the final version for publication. **SN** contributed to the conception and design of the studies, analyses and data interpretation, drafting the manuscript, and has approved the final version for publication. **KB** contributed to the conception and design of the studies, analyses and data interpretation, drafting the manuscript, and has approved the final version for publication. **DEcIDE Patient Outcomes In End Stage Renal Disease Study Authors** contributed to the conception and design of the studies, analyses and data interpretation, drafting the manuscript, and have approved the final version for publication. All authors read and approved the final manuscript

## Authors’ information

DEcIDE Patient Outcomes In End Stage Renal Disease Study Authors Include: Arrigain S (arrigas@ccf.org), Collins AJ (acollins@cdrg.org), Cook C (ccook17@jhmi.edu), Coresh J (jcoresh@jhsph.edu), Gilbertson DT (dgilbertson@cdrg.org), Guo H (hguo@cdrg.org), Herzog CA (cherzog@cdrg.org), Jolly SE (jollys@ccf.org), Lui X (LiuX@ccf.org), Luly J (jluly1@jhmi.edu), McDermott A (amcdermo@jhsph.edu), Meyer KB (kmeyer@tuftsmedicalcenter.org), Michels WM (W.M.Michels@amc.uva.nl), Nally JV Jr. (nallyj@ccf.org), Plantinga L (laura.plantinga@emory.edu), Scheel PJ (pscheel1@jhmi.edu), Schold JD (ScholdJ@ccf.org), Zager PG (pzag@unm.edu), Zhou J (jzhou9@jhmi.edu).

## Pre-publication history

The pre-publication history for this paper can be accessed here:

http://www.biomedcentral.com/1471-2369/13/167/prepub

## Supplementary Material

Additional file 1 Appendix. Click here for file
