# Supplementary material for: Comparative effectiveness studies to improve clinical outcomes in end stage renal disease: the DEcIDE patient outcomes in end stage renal disease study
Source: BMC Nephrol. 2012 Dec 6;13:167. doi: 10.1186/1471-2369-13-167 (PMC3554422; doi:10.1186/1471-2369-13-167)
Supplement: Additional file 1 — Appendix. [file 1471-2369-13-167-S1.doc]

Appendix

A.1. A. Comorbidity

Comorbidity will be assigned based on data from the CMS ESRD Medical Evidence Report Medicare Entitlement and/or Patient Registration (Form CMS-2728) and ICD9 codes from Medicare claims data according the procedures described by Liu et. al.^1^ A patient is defined as having a comorbid condition if a code for it can be found on the Medical Evidence Report or in claims from the date of dialysis initiation to time of follow-up. Comorbidity is assessed for each at 30 day intervals from date of initiation until the end of observation period.

A.1.1. Medical evidence report

Comorbid conditions will be identified from the primary cause of ESRD as well as the list of comorbid conditions selected on the form for conditions identified for the analyses (e.g. amputation, atherosclerotic heart disease, malignant neoplasm, cancer, congestive heart failure, chronic obstructive pulmonary disease, cerebrovascular disease, transient ischemic attack, diabetes, diabetic retinopathy, history hypertension, peripheral vascular disease).

A.1.2. Medicare claims data

Comorbid conditions will be identified from claims data using methods described by Herbert et. al. that utilize hospital outpatient, physician/supplier, skilled nursing facility, home health agency, and inpatient hospitalization claims data.2 Comorbid conditions from claims are defined using ICD-9 CM codes. The ICD-9 CM diagnosis codes and V codes defining the conditions listed in the table below:

| **Comorbid Condition** | **ICD9-CM diagnosis codes** | **ICD9-CM V codes** |
| --- | --- | --- |
| Atherosclerotic heart disease | 410–414 | V45.81;V45.82 |
| Congestive heart failure | 398.91; 422; 425; 428; 402.01; 402.11; 404.01; 404.11; 404.91; 404.03; 404.13; 404.93 | V42.1 |
| Cerebrovascular accident/transient ischemic attack | 430–438 |  |
| Peripheral vascular disease | 440–444; 447; 451–453; 557 |  |
| Other cardiac | 420–421; 423–424; 429; 785.0–785.3 | V42.2; V43.3 |
| Chronic obstructive pulmonary disease | 491–494; 496; 510 |  |
| Gastrointestinal bleeding | 456.0–456.2; 530.7; 531–534; 569.84; 569.85; 578 |  |
| Liver disease | 570; 571; 572.1; 572.4; 573.1–573.3 | V42.7 |
| Dysrhythmia | 426–427 | V45.0;V53.3 |
| Cancer | 140–172; 174–208; 230–231; 233–234 |  |
| Diabetes | 250; 357.2; 362.01-362.07; 366.41 |  |

Weights are assigned to each comorbid condition as follows: a weight of 1 is assigned to ASHD and diabetes; 2 to CVA/TIA, PVD, COPD, gastro-intestinal bleeding (GI), dysrhythmia, other cardiac disease, liver disease, and cancer; and 3 to CHF. The comorbidity score for each patient is defined as the sum of the weights based on presence or absence of the conditions.

A.2. B. Hospitalizations

Hospitalization events will be identified through Medicare Institutional Claims data.

A.2.1. Assessment of cardiovascular disease hospitalizations

Cardiovascular disease (CVD) hospitalizations will be assigned as the underlying cause of hospitalization in DEcIDE ESRD Outcomes study using ICD9-CM and CPT codes as defined in the table below. The event is considered to have occurred if any of the criteria are met (e.g. ICD-CM primary diagnosis code, ICD9-CM secondary diagnosis code, or CPT code).

| **Definition or Term** | **ICD9-CM Primary Diagnosis Codes** | **ICD9-CM Secondary Diagnosis Codes** | **CPT Codes** |
| --- | --- | --- | --- |
| Congestive heart failure (CHF), Cardiomyopathy | 398.91, 402.01, 402.11, 402.91, 404.01, 404.03, 404.11, 404.13, 404.91, 404.93, 425, 428, 518.4, 276.6 |  |  |
| Ischemic heart disease (IHD) | 410-414 | 410, 411.81 | 36.0, 36.9, 36.1, 36.2 |
| Conduction disorders and dysrhythmias | 426-427 798 | 427.4, 427.41-427.42, 427.5 | 37.6-37.8, 37.94-37.97, 00.50-00.54 |
| Cerebrovascular disease | 430-438 | 430, 431, 432, 433.01, 434.01, 436, 437.2, 435–435.9 | 38.10, 38.11, 38.12 |
| Circulatory system | 440-456, 458–459, 250.7 |  | 38.13,38.18, 38.38,39.22, 39.25,39.25, 39.26,39.28, 39.29, 84.0, 84.1, 84.91 |
| Other cardiac | 394-398, 415–420, 422–424, 429 |  |  |

A.2.2. Assignment of infectious hospitalizations

Infectious hospitalizations will be assigned using ICD-9 CM codes using both Medicare institutional and provider/supplier claims data as defined in the table below. Additionally:

• Primary diagnosis code of infection defined in the table below.

• If primary code is sepsis, check for any secondary code that is access related infection.

| **Category** | **ICD-9 CM Diagnosis Codes** |
| --- | --- |
| Sepsis | 038, 790.7 |
| Access related infection | 996.62, 567, 996.68, 999.31 |
| Endocarditis | 036.42, 421 |
| Respiratory Tract Infection | 011, 012, 033, 034, 460–466, 473, 480–488, 490, 513, 491.1, 494, 510–511, 513.0, 518.6, 519.01, |
| Skin/bone/joint infections | 015, 035, 036.82, 730, 680–686.9, 711, 996.66, 996.67, 706.0 |
| Gastrointestinal lumen infections | 001, 002, 003,004, 005, 006, 007, 008, 009, 567, 014, 569.5 |
| Central nervous system infections | 036.0, 036.1, 036.2, 047, 049, 320, 324, 013, 036.81, 045–049, 062, 063, 064, 320-326 |
| Fungal infections | 112, 117 |
| Genitourinary infections | 016, 590–590.9, 595–595.4, 597–597.89, 598.0, 599.0, 601–601.9, 604–604.9, 607.1, 607.2, 608.0, 608.4, 614–616.1, 616.3-616.4, 616.8, 670, 996.64, 996.65, |
| Other | 010, 017, 018, 020–027, 030, 031, 032, 036.3, 036.4,036.40, 036.41, 036.43, 036.89, 036.9, 037, 039, 040, 041, 042, 050–059, 060, 061, 065, 066, 070–079, 080–088, 090–099,100-139, 254.1, 331.81, 372–372.39, 373.0-373.2, 388.60, 382–382.4, 383.0, 386.33, 386.35, 390–393, 422.0, 422.91-422.93, 472–474.0, 475–476.1, 478.21-478.24, 478.29, 522.5, 522.7, 527.3, 528.3, 540–542, 566, 572–572.1, 573.1-573.3, 575–575.12, 611.0, 790.8, 996.60, 996.61, 996.63, 996.69, 997.62, 998.5, 999.3 |

A.3. C. Mortality

Mortality will be assigned as the cause of death through use of data from the National Death Index (NDI) records. Patient deaths will be ascertained through data from the ESRD Death Notification (Form CM-2746). NDI records will be obtained for each patient death through linkage with data from the USRDS. These records provide cause of death information (ICD-10 codes) from the U.S. Standard Certificate of Death. All codes in Section 27, Part I (including immediate and underlying causes of death) will be included. Cause of death will be assigned using only the primary cause of death. If the primary cause is a renal related code (e.g. diabetes with kidney complications [E08.2-E02.29, E09.2-E0.29, E10.2-E10.29, E11.2-E11.29, E13.2-13.29], hypertensive kidney disease [I12-I12.9, I13-I13.2], intrinsic renal disease [N00-N08, N10-N19, N29, N28], small kidney of unknown cause [N27], unspecified contracted kidney [N26], congenital malformations of the urinary system [Q60-Q64]), cause of death will be assessed using the secondary cause of death code.

A.4. Cause specific mortality

A.4.1. Cardiovascular disease related mortality

Primary cause of death due to cardiovascular disease will be assessed using the codes as outlined in the table below:

| **Category** | **ICD-10 Diagnosis Codes** |
| --- | --- |
| Chronic Rheumatic Heart Disease | I05-I09 |
| Hypertensive Heart Disease | I10, I11, I15 |
| Ischaemic Heart Disease | I20-I25 |
| Other Heart Disease | I33-I37, I42-I51 |
| Cerebrovascular Disease | I60-I69 |
| Disease of Arteries, Arterioles, and Capillaries | I70-I74, I77-I79 |
| Other and unspecified Circulatory Systems | I98-I99 |
| Vascular Disorders of Intestine | K55 |
| Atherosclerotic Vascular Disease |  |
| Coronary Artery Disease | I21, I22, I24, I25.0, I25.1, I25.5, I25.6, I25.8, I25.9, I51.6 |
| Cerebrovascular Disease | I61, I62.9, I63, I64, I67.2, I67.9 |
| Abdominal Aortic Aneurysm | I71 |
| Peripheral Vascular Disease | I73.9, R02 |
| Ischemic Bowel Disease | K55 |

A.4.2. Infectious mortality

Primary cause of death due to infectious cause will be assessed using the codes as outlined in the table below:

| **Category** | **ICD-10 Diagnosis Codes** |
| --- | --- |
| Overall—Infectious Deaths codes | A00-B99, E06.0, G00-G08, I00-I01, I33, I38, J00-J06, J10-J18, J20-22, J36, J39.0, J39.1, J44.0, J85-J86, K35, K63.0, K65.0, K65.9, K80.0, K80.3, K80.4, K81, K83.0, L00-L08, M00-M02, M86, N30.0, N30.8, N41.0, N41.2, N41.3, O03.0, O03.5, O04.5, O07.0, O08.0, O75.3, O85-O86, R57.2, T80.2, T81.4, T82.6-T82.7, T83.5-T83.6, T84.5-T84.7, T85.7, T87.4, T88.0 |
| Sepsis/Bacteremia | A40-A41, O85 |
| Endocarditis | I33, I38, T82.6 |
| Respiratory Tract Infection | J00-J06, J09-J18, J20-22, J36, J39.0, J39.1, J44.0, J85-J86 |
